# A penta-substituted pyridine alkaloid from the rhizome of *Jatropha elliptica* (Pohl) Muell. Arg. is active against *Schistosoma mansoni* and *Biomphalaria glabrata*

**DOI:** 10.1007/s00436-013-3743-2

**Published:** 2014-02-06

**Authors:** Aldenir Feitosa dos Santos, Saskya Araújo Fonseca, Fernanda Andrade César, Mônica Camelo Pessoa de Azevedo Albuquerque, José Valfrido Santana, Antônio Euzébio Goulart Santana

**Affiliations:** 1 Grupo Estudo de Compostos Naturais Bioativos, Centro Universitário Cesmac, 57051-160 Maceió, AL Brazil; 2 Grupo de Pesquisa em Química, Universidade Estadual de Alagoas, 57312-270 Arapiraca, AL Brazil; 3 Laboratório de Imunologia e Esquistossomose Experimental, Laboratório de ImunopatologiaKeizoAsami, Universidade Federal de Pernambuco, 50670-901 Recife, PE Brazil; 4 Laboratório de Pesquisa em Recursos Naturais, Instituto de Química e Biotecnologia, Universidade Federal de Alagoas, 57092-970 Maceió, AL Brazil

## Abstract

*Jatropha elliptica* is a shrub distributed throughout the north and west of Brazil and reputedly possesses a wide range of therapeutical properties. The roots of this plant possess molluscicidal activity and contain terpenoids, coumarin, lignoid, steroids and alkaloid. In the present study, we assessed the schistosomicidal, miracicidal and cercaricidal activities (against *Schistosoma mansoni*) and molluscicidal activities (against adults and egg masses of *Biomphalaria glabrata*) of the alkaloid diethyl 4-phenyl-2,6-dimethyl-3,5-pyridinedicarboxylate, isolated from the ethanol extract of the rhizome of *J. elliptica*, have been determined. The alkaloid was 100 % lethal to adult schistosomes within 4 days at a concentration of 50 μg/mL. Alterations were observed in the schistosome tegument occasioned by treatment with the alkaloid, such as formation of vesicles and vacuolisation. The extent of tegumental damage of the worm was proportional to the time of incubation and to the concentration of compound. The alkaloid also exhibited a potent cercaricidal activity (LC_100_ = 2 μg/mL); it was totally ineffective against miracicidal forms of the parasite. Moreover, the alkaloid presented strong activity against adult snails (LC_90_ = 36.43 μg/mL) but was inactive against their egg masses. It is observed then the potential of this compound for the development of new therapies for the treatment of schistosomiasis.

## Introduction

Schistosomiasis is the second most prevalent tropical disease affecting more than 200 million people worldwide (WHO 2005; Rollemberg et al. 2011). Moreover, the prevalence of this disease is increasing in many areas of the world owing to the poor level of basic sanitation and the low social and economic status of the respective populations. In Brazil, eight million people from endemic regions stretching from the north to the south-east of the country are infected with this chronic debilitating disease (Katz and Peixoto 2000).

The two main approaches for the control of schistosomiasis currently involve reducing the transmission of the disease and applying chemotherapy in identified cases, and this situation is unlikely to change until a suitable vaccine becomes available.

Praziquantel is currently the drug of choice for the treatment of schistosomiasis. However, this drug does not prevent reinfections, is inactive against juvenile schistosomes and has a limited effect as well on already developed liver and spleen lesions. In addition, there is a considerable concern about the development of praziquantel resistance of some strains. These reports emphasise the need of developing new schistosomicidal drugs for the treatment of this neglected tropical disease (Magalhães et al. 2009, 2010; Miranda et al. 2012).

One strategy that has been used to regulate schistosomiasis is through control of the mollusc that acts as the intermediate host. Molluscicides currently in use are typically synthetic compounds (copper sulphate or carbonate, niclosamide and pentachlorophenate), although a number of agents of natural origin have been reported (Luna et al. 2005; Shukla et al. 2006). Control of schistosomiasis using a molluscicide would be far more effective if the agent also possessed miracidicidal and cercaricidal activities. Although both miracidia and cercariae are short-lived (lifespan of 12–48 h), their elimination is not trivial. In the case of cercariae, for example, multiple treatments of infested sites are necessary since infected molluscs continually shed new cercariae into the water.

The use of drugs derived from plants, fungi, bacteria and marine organism has a long tradition in medicine. Plants constitute a rich source of bioactive chemicals. In addition, herbal medicines are generally more accessible and affordable and are an important part of the culture and traditions of many populations (Pontin et al. 2008; Koné et al. 2012). The interest in medicinal plants as new sources of antiparasitic drugs is rising (Ferreira et al. 2011). In the past years, many studies have been undertaken to assess the potential of medicinal extracts against parasitic diseases (Elango and Rahuman 2011; Ghosh et al. 2011).

Various natural compounds have been shown to be toxic to miracidia and cercariae (Santos et al. 2007; Kamel et al. 2010), and some plants were reported to possess components that can inhibit the penetration of cercariae through human skin (De-Carvalho et al. 1998). Crude aqueous extract of *Zingiber officinale* (Mostafa et al. 2011) and pure compounds as artemether (Abdul-Ghani et al. 2011) and dihydroartemisinin (Li et al. 2011) have showed in vivo schistosomicidal effects, but so far, no new drug has been marketed for the treatment of schistosomiasis (Miranda et al. 2012).

**Fig. 1:**
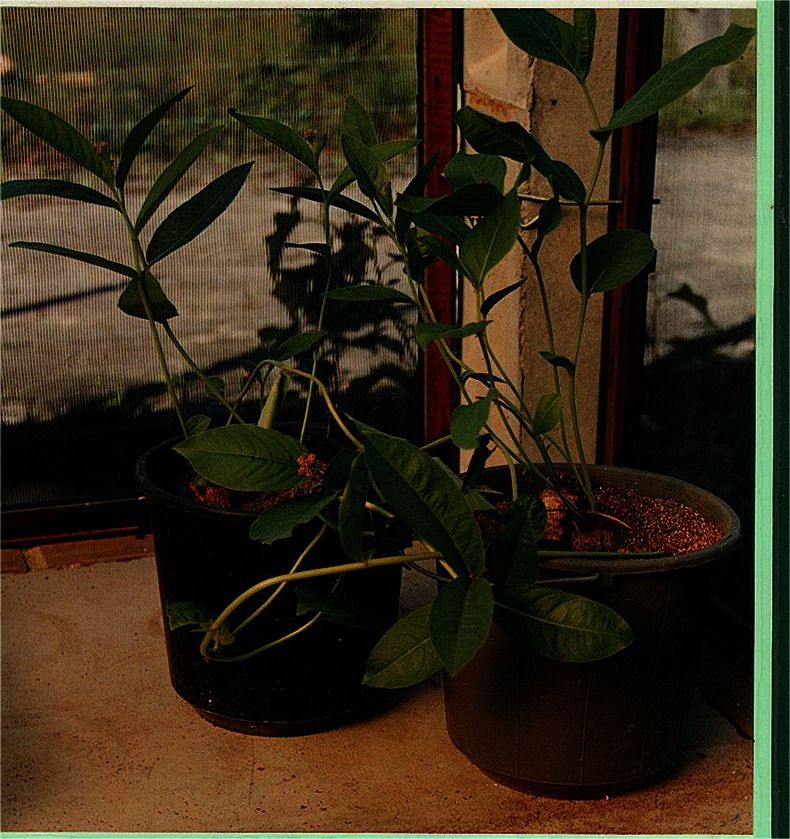
*Jatropha elliptica* (Pohl) Muell Arg. cultivated in greenhouse

**Fig. 2:**
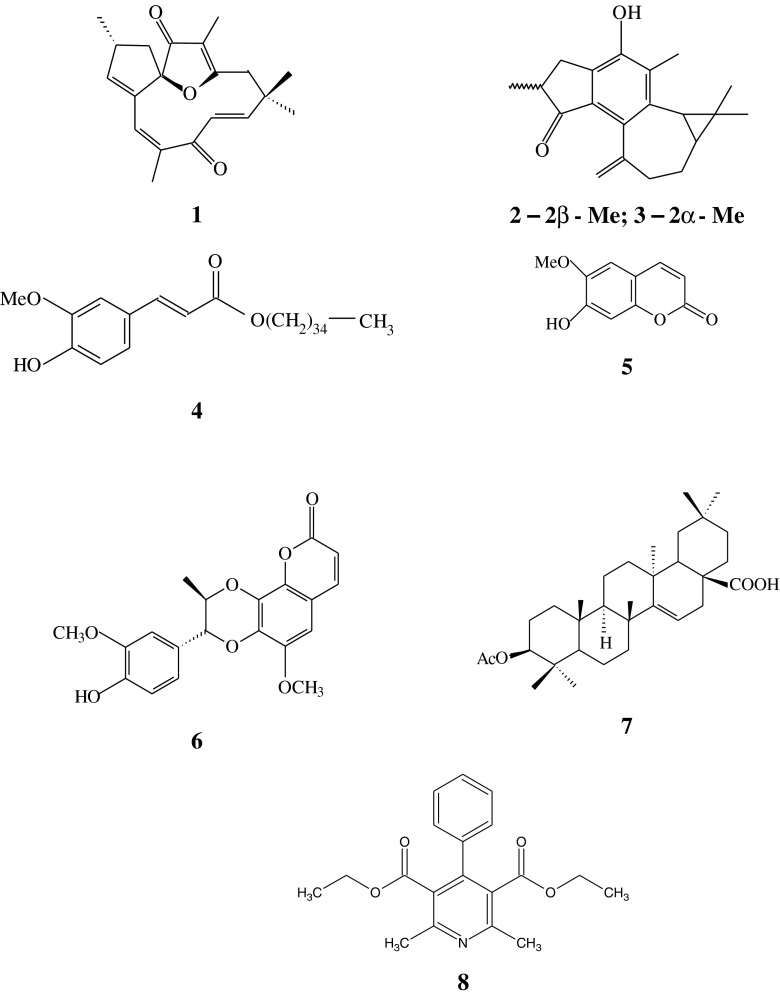
Compounds isolated from ethanol extract of the rhizome of *J. elliptica*

During our search of the Brazilian flora for extracts and compounds that could be used to control *Biomphalaria glabrata*, we have been able to demonstrate that the roots of *Jatropha elliptica* (Pohl) Muell Arg. (Euphorbiaceae) possess molluscicidal activity (Santos and Sant’Ana 1999). *J. elliptica* is an annual shrub (Fig. 1) distributed throughout the north and west of Brazil and reputedly possesses a wide range of therapeutical properties (Lima et al. 2006; Sabandar et al. 2013). The diterpenoids jatrophone (1) and jatropholones A and B (2 and 3), the ester pentatriacontanyl ferulate (4), the coumarin fraxetin (5), the coumarin lignoid propacin (6), the triterpenoid acid 3-*O*-acetyl aleuritolic acid (7) and a mixture of steroids β-sitosterol and stigmasterol have been previously isolated from the rhizome of *J. elliptica* (Goulart et al. 1993). More recently, a penta-substituted pyridine, namely diethyl 4-phenyl-2,6-dimethyl-3,5-pyridinedicarboxylate (8) has been identified in the roots of the plant (Fig. 2) (Marquez et al. 2005; Santos et al. 2005).

In the present paper, we report the evaluation of schistosomicidal, miracidicidal or cercaricidal activities against *Schistosoma mansoni* and/or molluscicidal activity against *Biomphalaria glabrata* of the alkaloid diethyl 4-phenyl-2,6-dimethyl-3,5-pyridinedicarboxylate (penta-substituted pyridine alkaloid) isolated from the ethanol extract of the rhizome of *J. elliptica*.

## Material and methods

### Plant material, extraction and fractionation

Roots of *J. elliptica* Muel. Arg. were collected in the state of Goiás, Brazil. Plant material was authenticated by Professor José Elias de Paula and a voucher specimen [JEP 1863 (UB)] is deposited in the herbarium of Universidade de Brasilia, Brasilia DF. Powdered, air-dried root material was extracted and fractionated as described earlier (Santos et al. 2005).

### Snails and schistosomes

A colony of *B. glabrata* was maintained at the Departamento de Parasitologia (Universidade Federal de Pernambuco—UFPE, Recife, PE, Brazil) as described previously Santos and Sant’Ana 1999. Adult snails (shell diameters 19–25 mm) and egg capsules were employed in bioassay experiments. The BH strain (Belo Horizonte, MG, Brazil) of *S. mansoni* was routinely maintained at the Laboratório de Imunopatologia Keiko Asami (UFPE) by passage through *B. glabrata* and mice (El-Beshbishi et al. 2013).

### Preparation of working solutions for the bioassays

#### Adult schistosome bioassay

A stock solution (400 μg/mL) was prepared for each sample by dissolving an aliquot (8 mg) in dimethyl sulphoxide (DMSO; 200 μL) and adding RPMI-1640 medium to a final volume of 20 mL. Appropriate aliquots of the stock solution were diluted with parasite culture medium to yield concentrations of 200, 100, 50 and 10 μg/mL. Six replicates of each bioassay were carried out for each sample concentration (Mohamed et al. 2005).

#### Miracidia bioassay

A stock solution (1 mg/mL) was prepared for each sample by dissolving an aliquot (15 mg) in DMSO (150 μL) and adding dechlorinated water to a final volume of 15 mL. Samples (150 μL) were typically assayed in duplicate at concentrations of 1, 10 and 100 μg/mL (Mohamed et al. 2005).

#### Cercariae bioassay

A stock solution (200 μg/mL) was prepared for each sample by dissolving an aliquot (3 mg) in DMSO (150 μL) and adding dechlorinated water to a final volume of 15 mL, as previously described (Santos et al. 2007).

#### Adult snail bioassay

Stock solutions for bioassays were prepared according to a previously described protocol (Santos et al. 2010).

#### Egg mass bioassay

Test solutions were prepared according to a previously described protocol (Santos and Sant’Ana 2001).

### Protocols for the bioassays

#### Schistosomicidal activity

Strict aseptic techniques were employed throughout the experiments Mercer and Chapell (1985). Two adult schistosomes, paired or unpaired, in parasite culture medium were placed into one well of a multi-well plastic tissue culture plate, an appropriate aliquot of sample solution was added, and the plates were incubated at 37 ± 1 °C in a humid atmosphere containing 5 % CO_2_. The viability of the worms was observed under an inverted microscope each day until the fifth day after sample addition. Schistosomes were considered dead when no movement could be detected over a 3-min observation period. Positive controls (10 μg/mL praziquantel [Sigma]) and negative controls (RPMI-1640 medium containing 1 % *v*/*v* DMSO) were included in every experiment, and a minimum of 30 schistosomes were employed in the assay of each treatment and control group.

#### Miracidicidal activity

Twenty miracidia were exposed to each concentration of a sample in multi-well plates as described previously (Mohamed et al. 2005) together with positive controls (0.1 μg/mL niclosamide) and negative controls (dechlorinated water containing 1 % *v*/*v* DMSO).

#### Cercaricidal activity

Approximately 500 freshly released cercariae suspended in dechlorinated water were placed into a 25-mL beaker and an appropriate aliquot of sample solution was added as previously described (Santos et al. 2007).

#### Molluscicidal activities

Activities against adult snails and egg masses were evaluated according to established procedures (Santos and Sant’Ana 2001; Santos et al. 2010; Teixeira et al. 2012).

### Statistical methods

LC_10_, LC_50_ and LC_90_ values, together with their 95 % confidence intervals, were determined through probit analysis of the mortality data (Finney 1971). In those cases in which the data were insufficient to calculate a 95 % confidence interval, the lethal concentration was determined via logit transformation (Hafner et al. 1977).

## Results

### Bioactivity-directed fractionation

An ethanolic extract of the rhizome of *J. elliptica*, which had previously been shown to exhibit molluscicidal activity against *B. glabrata*, has been subjected to fractionation guided by activity against cercariae of *S. mansoni* (Table 1). Fractions containing compound 8 presented significant cercaricidal activities, and this component was isolated and identified as diethyl 4-phenyl-2,6-dimethyl-3,5-pyridinedicarboxylate.

**Table 1:** Bioactivity-directed fractionation of an ethanolic extract of *J. elliptica* according to cercaricidal activity against *S. mansoni*

Additive	Additive concentration (μg/mL)	Inhibition^a^ of cercaria after			
		15 min	30 min	1 h	2 h
Ethanolic extract	100	−	±	+	+
	95	−	±	+	+
	90	−	−	−	−
Ethyl acetate fraction	100	−	−	+	+
	10	−	−	+	+
	1	−	−	−	−
Fraction 16–21^b^	100	−	+	+	+
	10	−	+	+	+
	1	−	−	−	+
Compound 8^c^	100	+	+ +	+ +	+ +
	10	−	±	±	+
	1	−	−	−	+

^a^(+ +) 100 % of larvae motionless at the bottom of the test tube, (+) ≅90 % of larvae motionless at the bottom of the test tube, (±) ≥50 % of larvae motionless and/or at the bottom of the test tube and (−) lack of larvicidal activity with >90 % of larvae swimming

^b^Fractions obtained following column chromatography

^c^Diethyl 4-phenyl-2,6-dimethyl-3,5-pyridinedicarboxylate recrystallised from acetone

### Schistosomicidal activity in vitro

Table 2 shows that the in vitro exposure of *S. mansoni* adult worms to 8 resulted in the inhibition of movement and death, effects that were concentration dependent (Table 2). Following 4 days of exposure to 8 at a concentration of 50 μg/mL, 100 % of adult schistosomes were observed to be dead and unpaired compared with control pairs that had been treated with solvent (RPMI 1640 medium containing 1%DMSO) alone. Alterations to the schistosome tegument occasioned by treatment with 8 could be readily observed under the microscope and were manifested in the formation of vesicles that became apparent as early as 24 h after the introduction of the additive. Continued exposure led to vacuolisation followed by severe tegumental damage; the extent of which was proportional to the time of incubation and to the concentration of additive.

**Table 2:** In vitro effects of diethyl 4-phenyl-2,6-dimethyl-3,5-pyridinedicarboxylate (compound 8) isolated from the ethanol extract of the rhizome of *J. elliptica* against 56-day-old adult *S. mansoni*

Group	Incubation of period (h)	Dead worms %	Motor activity reduction (%)		Worms with tegumental alterations (%)	
			Slight	Significant	Parcial	Extensive
Control^a^	24	0	0	0	0	0
	48	0	0	0	0	0
	72	0	0	0	0	0
	96	0	0	0	0	0
	120	0	0	0	0	0
Praziquantel 10 μg/mL	24	100	0	100	0	0
	48	100	0	100	0	100
	72	100	0	100	0	100
	96	100	0	100	0	100
	120	100	0	100	0	100
Compound 8 10 μg/mL	24	0	0		0	0
	48	0	0		0	0
	72	0	100		0	0
	96	0	25	75	0	0
	120	0	0	100	0	0
50 μg/mL	24	0	0		0	0
	48	0	10	20	0	0
	72	0	10	80	0	0
	96	100	0	100	0	0
	120	100	0	100	0	0
100 μg/mL	24	0	0	0	0	0
	48	65	0	100	100	0
	72	85	0	100	100	0
	96	100	0	100	0	100
	120	100	0	100	0	100
200 μg/mL	24	100	0	100	0	100
	48	100	0	100	0	100
	72	100	0	100	0	100
	96	100	0	100	0	100
	120	100	0	100	0	100

^a^RPMI 1640 + 1 % DMSO

### Miracicidal and cercaricidal activity

Compound 8 exhibited a significant concentration-dependent activity against *S. mansoni* cercariae but was totally inactive against the miracidia (Table 3). At a concentration of 4 μg/mL, 8 completely immobilised cercariae after an exposure time of 30 min.

**Table 3:** Activity of diethyl 4-phenyl-2,6-dimethyl-3,5-pyridinedicarboxylate (8) against miracidia and cercariae of *S. mansoni*

Larvae	Additive (concentration μg/mL)	Inhibition^a^ of larvae after				LC_100_ (μg/mL)
		15 min	30 min	1 h	2 h	
Miracidia	VIII (100)	−	−	−	−	Inactive
	VIII (10)	−	−	−	−	
	VIII (1)	−	−	−	−	
	Niclosamide (3)	++	++	++	++	Active
	Dechlorinated water containing 1 % DMSO	−	−	−	−	Inactive
Cercariae	VIII (8)	+	+ +	+ +	+ +	2.0
	VIII (6)	+	+ +	+ +	+ +	
	VIII (4)	+	+ +	+ +	+ +	
	VIII (2)	±	+	+	+	
	VIII (1.5)	−	−	−	−	
	VIII (1.0)	−	−	−	−	
	Niclosamide (3)	++	++	++	++	Active
	Dechlorinated water containing 1 % DMSO	−	−	−	−	

^a^(+ +) 100 % of larvae motionless at the bottom of the test tube, (+) ≅90 % of larvae motionless at the bottom of the test tube, (±) ≥50 % of larvae motionless and/or at the bottom of the test tube and (−) lack of larvicidal activity with >90 % of larvae swimming

### Molluscicidal activity

A preliminary screen indicated that 8 was totally inactive against snail egg masses even at a concentration of 100 μg/mL whilst, in the same bioassay, copper carbonate at 50 μg/mL was 100 % lethal. In contrast, detailed assays of the activity against adult snails of 8 in the concentration range 10–50 μg/mL were performed, and the LC_10_, LC_50_ and LC_99_ values was determined through probit analysis (Table 4).

**Table 4:** Bioactivity of diethyl 4-phenyl-2,6-dimethyl-3,5-pyridinedicarboxylate (8) against *B. glabrata* adults

Additive	Diameter of snails (mm)	Concentration (μg/mL)	Mortality (%)	LC_10_ (μg/mL)^a^	LC_50_ (μg/mL)^a^	LC_90_ (μg/mL)^a^
Compound 8^b^	17–24	50	100	7.57 CI_95_ = 4.57–9.97	16.60 CI_95_ = 13.52–19.59	36.43 CI_95_ = 29.45–52.21
		30	83			
		20	50			
		10	26			
Copper carbonate	21–24	50	100			
Dechlorinated water containing 0.1 % DMSO	21–24		0			

^a^Determined after 96 h incubation

^b^Diethyl 4-phenyl-2,6-dimethyl-3,5-pyridinedicarboxylate

## Discussion

This study was performed to evaluate in vitro the anti-schistosomal effect of penta-substituted pyridine 8 for controlling schistosomiasis. In the last decades, plant extracts were widely used for the treatment of schistosoma infection (Molgaard et al. 2001).

The penta-substituted pyridine 8 has already been reported to possess strong multidrug resistance reversal activity (Marquez et al. 2005). In the present study, 8 was assayed for potential schistosomicidal, miracidicidal, cercaricidal and molluscicidal properties.

The lethal concentration against *S. mansoni* of 8 was fourfold lower than that of the quassinoid longilactone isolated from the leaves of *Eurycoma longifolia* (Jiwajinda et al. 2002). This compound lead to the separation of all couple worms and extensive disruption on their teguments, such as sloughing. Similar results were presented by solasonine (50 μM), solamargine (32 and 50 μM) and equimolar mixture of glycoalkaloids (20, 32 and 50 μM) steroidal alkaloids from *Solanum lycocarpum* fruits (Miranda et al. 2012). The action on the tegument is important since this protects the schistosome against attack by the immune system of the host and is also involved in nutrient absorption and secretory functions; it is considered to be an important target for anti-schistosomal drugs (Utzinger et al. 2000).

Studies with phloroglucinol compounds, obtained from the rhizomes of *Dryopteris* species against *S. mansoni* adult worms, also showed similar results, but with a faster action. All worm pairs were dead after 24 h of incubation with aspidin 25 to 100 μM, flavaspidic acid 50 and 100 μM, methylene-*bis*-aspidinol 100 μM and desaspidin 25 to 100 μM (Magalhães et al. 2010).

Infection with *S. mansoni* occurs when the cercaria penetrates the body of man through intact skin. Therefore, to prevent such penetration is also a potential way of controlling infection (De-Carvalho et al. 1998). Activity presented by compound 8 was considerably stronger than that presented by the mixture of robustic acid, alpinum isoflavone and dimethylalpinum isoflavone isolated from the seeds of *Milletia thonningii*, which was lethal to cercariae at a concentration of 50 μg/mL after an exposure time of 30 min (Lyddiard et al. 2002). In contrast, however, the synthetic molluscicide niclosamide when applied at a concentration of 0.05 μg/mL induced 87 and 69 % mortality, respectively, in cercariae and miracidia of *S. mansoni* following 2 h of exposure (De-Carvalho et al. 1998).

Whilst *Biomphalaria* spp. control by molluscicides is one of the main strategies to reduce the snail population in infected areas, there are few effective molluscicides commercially available. Therefore, natural products may be considered as potentially useful and safe molluscicides (Miyasato et al. 2012). In this research, the compound 8, in comparison with the main components of the ethanolic extract of *J. elliptica* by Santos and Sant’Ana (1999), showed an activity (LC_90_ = 36.43 μg/mL) lower than that of jatrophone (LC_90_ = 8.91 μg/mL) but much higher than those of the jatropholones A and B (LC_90_ = 206.16 μg/mL). The molluscicidal activity of 8 is significantly lower than those of niclosamide (LC_100_ = 1.5 μg/mL), which is recommended by the World Health Organization (WHO 1993) for large scale use in schistosomiasis is control programmes.

## Conclusion

Currently, chemotherapy is the most widely employed method for the control of schistosomiasis, although treatment is based on just a few available drugs. Recently, a diminished response to praziquantel (the drug of choice for the treatment of the disease) has been reported suggesting the appearance of non-susceptible strains of the parasite. For this reason, the search for new anti-schistosomal drugs, particularly those deriving from natural sources, has attained a greater importance. In this context, the present study has demonstrated that diethyl 4-phenyl-2,6-dimethyl-3,5-pyridinedicarboxylate (8), derived from the rhizome of *J. elliptica*, is highly active against adult *S. mansoni* and free-living cercariae and against the adult snail host *B. glabrata*. In contrast, 8 was inactive against the miracidia of the schistosome and against egg masses of the snail. However, further studies, including in vivo assays, are necessary to fully determine the potential of this compound for the development of new therapeutics to treat schistosomiasis.
